# Design and Performance Analysis of an Ultrasonic System for Health Monitoring of Concrete Structure

**DOI:** 10.3390/s21196606

**Published:** 2021-10-03

**Authors:** Jinhui Zhao, Tianyu Hu, Ruifang Zheng, Penghui Ba, Qichun Zhang

**Affiliations:** 1 Key Laboratory for Technology in Rural Water Management of Zhejiang Province, College of Electrical Engineering, Zhejiang University of Water Resources and Electric Power, Hangzhou 310018, China; 2 School of Mechanical and Electrical Engineering, China Jiliang University, Hangzhou 310018, China; s1801081103@cjlu.edu.cn (T.H.); zheng_ruifang4716@163.com (R.Z.); p1901085201@cjlu.edu.cn (P.B.); 3 Department of Computer Science, University of Bradford, Bradford BD7 1DP, UK; q.zhang17@Bradford.ac.uk

**Keywords:** nondestructive detection of concrete structure, ultrasonic instrument, data acquisition, performance analysis

## Abstract

The development and research of an ultrasonic-based concrete structural health monitoring system encounters a variety of problems, such as demands of decreasing complexity, high accuracy, and extendable system output. Aiming at these requirements, a low-cost extendable system based on FPGA with adjustable system output has been designed, and the performance has been evaluated by different assessment parameters set in this paper. Besides the description of the designed system and the experiments in air medium, the residual similarity and Pearson correlation coefficients of experimental and theoretical data have been used to evaluate the submodules’ output. The output performance of the overall system is evaluated by the Pearson correlation coefficient, root-mean-square error (RMSE), and magnitude-squared coherence with 40 experimental data. The maximum, median, minimum, and mean values in three-parameter datasets are analyzed for discussing the working condition of the system. The experimental results show that the system works stably and reliably with tunable frequency and amplitude output.

## 1. Introduction

At present, there are many ways to detect the health condition of concrete, among which the most widely used method is the ultrasonic testing method [1]. Concrete is a complex, multi-phase, and heterogeneous medium, and a series of complex acoustic phenomena will be generated when using ultrasonic to detect concrete. Ultrasonic propagates in concrete with quick attenuation and a lot of noise, making it difficult to obtain accurate, stable, and clear detection waveforms [2,3]. Therefore, a stable and reliable ultrasonic testing system is an important basis for analyzing the health condition of concrete.

A concrete ultrasonic testing instrument is generally composed of a high-voltage pulse signal generator, an amplifier, and sensors [4,5]. Existing instruments, such as the American NDT James concrete ultrasonic testing device, have insufficient expansibility and flexibility, complex structure, and low resolution. W. Liu et al. [6] used the single-chip AT89C52 as the control core of the ultrasonic flaw detector, using an untuned transmitter circuit to generate a wideband narrow pulse to excite the piezoelectric transducer and cascading AD603 as the operational amplifier circuit of the echo signal. However, its transmitting circuit is affected by the reciprocal voltage, and the received signal contains a lot of noise. V. Dumbrava et al. [7] used a half-bridge structure to design a pulse generator to excite the ultrasonic transducer. Its anti-imbalance ability is strong, but it has the disadvantages of low efficiency and power compared to the full-bridge structure. Scholars analyze the impacts of the sharp pulse and the square pulse on the excitation of ultrasonic probes and find that the square pulse can make the wafer produce higher vibration amplitude, and this is more suitable for high-power ultrasonic detection systems [8]. X.P. Wang et al. [9] used an FPGA-controlled full-bridge drive circuit to excite the sonar transducer for observing the amplitude and speed of the wave. The device has good ultrasonic emission performance. The receiving circuit mainly includes an amplifier and a filtering circuit, which has a better conditioning effect on the echo signal [10]. The application flexibility of the ultrasonic detection system constructed by FPGA is higher [11], and the 12-bit analog-to-digital conversion module can obtain high-precision detection signals [12]. A single-chip microcomputer, Complex Programming logic device (CPLD), and FPGA have been used in the ultrasonic testing system [13]. The price of FPGA is slightly higher than that of a single-chip microcomputer and CPLD, but FPGA runs faster, processes more complex data, and realizes more functions, which is conducive to improving the expansibility of the detection system. Therefore, FPGA is a cost-effective control core system.

Considering the advantages and disadvantages of these systems, it is necessary to design an open and extensible ultrasonic detection system for monitoring concrete health conditions. The system includes ultrasonic probes, an FPGA control core, a high-voltage full-bridge pulse transmitter module, and a receiving module that consists of an adjustable amplifier circuit, a filter circuit, and a high-speed sampling module. It has the functions of data acquisition, storage and transmission, and it also highlights the characteristics of adjustable output with a large dynamic transmission range. The designed system avoids complex structures and uses low-cost modules, which reduces the overall cost of the hardware equipment.

Only an ultrasonic detection system with excellent performance can accurately detect the health conditions of concrete. In detection technology, a series of indices including residual similarity, Pearson correlation coefficient, RMSE, and magnitude-squared coherence are used to evaluate the performance of the detection system. System performance assessment is used to process data to compare actual practical performance with benchmark work performance by timing analysis and to avoid abnormal operational status during subsequent usage through online or offline assessment. Some indices such as output variance and square correlation coefficient are used for the performance assessment [14]. Effectively evaluating the work performance of the designed and developed ultrasonic inspection system is a challenging task. Conclusions based on the relevant information of a performance assessment of the ultrasonic detection system show that there is no existing solution. However, there are related assessment and analysis methods of system performance in other application fields, such as the visible-light photoelectric detection system [15]. Moreover, in the credibility assessment of the simulation model, the consistency of the simulation output data and the actual data is evaluated to analyze the output performance of the simulation system [16]. These system assessment methods can be utilized for the performance assessment of the ultrasonic detection system partially.

In order to evaluate the working performance of the presented ultrasonic testing system, various assessment indices are introduced to analyze the experimental data to implement the performance assessment of the system. The residual similarity and Pearson correlation coefficient are used to quantitatively analyze the ultrasonic pulse waveform of the transmitter module in the ultrasonic detection system [17,18]. The working performance of the amplifier circuit and the filter circuit in the receiving module is evaluated by the amplification and filtering curve of the actual test data. Under the same conditions, 40 ultrasonic waveform data transmitted in the air are continuously collected, then the Pearson correlation coefficient, RMSE, and amplitude-squared coherence [19,20] are calculated to evaluate the continuous working stability of the ultrasonic detection system designed in this paper. The experimental results and their performance analysis prove that the designed system can work stably and reliably. Therefore, a simple and effective assessment method for the ultrasonic detection system is established to meet the requirement of performance analysis and evaluation and be helpful for signal processing and analysis of received ultrasonic waves. It should be noted that the analysis of the detection results tested on concrete is not included in this paper because the influence of ultrasonic propagation in concrete is complex and should be analyzed separately. Applications in concrete of the designed detection system, and research results of theoretical models of the ultrasonic detection system, will successively be presented and discussed separately.

The remainder of this article is organized as follows: In Section 2, we describe the presented ultrasonic detection system including the design and development. In Section 3, we evaluate the submodule performances of the system. In Section 4, we evaluate the performance of the overall system and prove that it is reliable. Finally, Section 5 presents our conclusions.

## 2. Description of the Ultrasonic Detection System

### 2.1. The Overall Design Scheme of the System

The ultrasonic detection system in this paper includes the main control module FPGA, ultrasonic transmitter module, signal receiver module, analog-to-digital conversion module, high-voltage DC power supply, ultrasonic probe, and PC terminal.

According to the frequency application range when using ultrasonic to detect concrete [21], this system adopts 50K-P28F 50 kHz ultrasonic probes. The ultrasonic transmission method has been used for detection.

For meeting the sampling requirements, a high-speed and high-precision analog-to-digital conversion chip and its peripheral circuits constitute the sampling module of the system. This module can accurately sample the ultrasonic detection signals, which are generated by the 50 kHz ultrasonic probe.

FPGA is a programmable signal processing device [22]. Compared with traditional digital circuit systems, FPGA has the advantages of being programmable and having high integration, high speed, and high reliability [23,24]. Altera’s FPGA (EP4CE15F23C8) as the control core of the system realizes effective control of the transmitter module and the fast transmission of received data.

**Figure 1:**
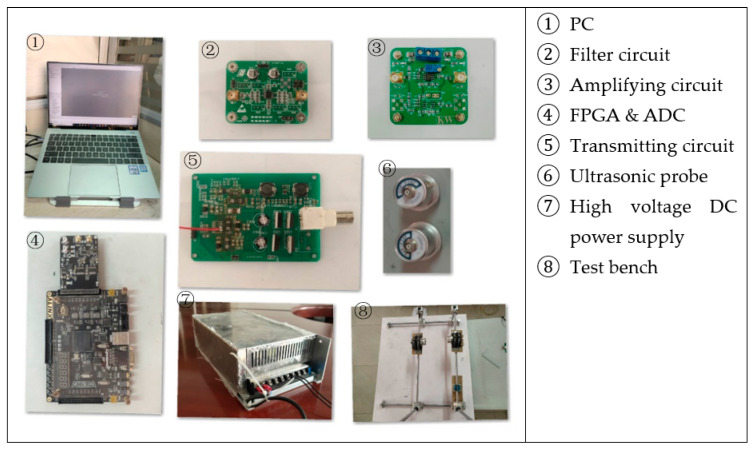
Physical maps of the testing system.

The ultrasonic transmitter module uses a full-bridge drive circuit to generate square wave pulses to excite the probe. The receiving module includes an operational amplifier and a filter circuit, used to amplify the detection signal and reject the high-frequency noise [25]. Figure 1 is a physical map including each part of the presented detection system in this paper.

### 2.2. Pulse Transmitting Module

The pulse transmitter module generates electrical pulses to excite the piezoelectric ceramic wafer of the ultrasonic probe to vibrate and form ultrasonic signals. The sharp pulse generated by the non-tuned transmitter circuit is one of the ways to excite the piezoelectric transducer [26]. The square wave pulse is generated by using the drive circuit to drive the MOS transistor on and off. Compared with the sharp pulse, the rise and fall times of the square pulse are shorter, the resolution of the amplitude is higher, and the stability of the output waveform is better [27,28]. The output power of the full-bridge circuit composed of MOS transistors is twice as large as that of the half-bridge circuit [29], which is more suitable for high-power applications. A bootstrap MOS transistor drive circuit is used for driving the full-bridge to excite the piezoelectric transducer.

Under the control of FPGA, the pulse transmitting circuit generates a square wave pulse signal with a certain width and a certain repetition frequency to drive the ultrasonic transducer. The DC switching power supply uses the step-down chip XD308H to stably step down the high-voltage DC power supply to the +9 V voltage required by the MOS transistor driver chip IR2110. XD308H is a non-isolated, high-voltage power supply step-down chip with ultra-wide range input that can adapt to the step-down requirements of different input voltages. It has the characteristics of no audio noise, low heat generation, etc., and it integrates comprehensive and complete protection functions. IR2110 has both optocoupler isolation and electromagnetic isolation, which can effectively prevent interference from the output end to the input end and can quickly respond to the control signal of the circuit. The driving circuit controls the turn-on and turn-off of the four MOS transistors IRFBC20 in the full-bridge through two IR2110 chips, thereby controlling the turn-on and turn-off of the adjustable DC power supply, forming a square wave pulse of unidirectional voltage or bidirectional voltage.

### 2.3. Signal Receiving Module

After the ultrasonic propagates in the concrete, there is a large attenuation, and the received signal is in millivolts or even microvolts with a lot of noise. To meet the requirements of ADC sampling, an operational amplifier module and a filter module are designed to amplify and filter the received signal. The operational amplifier module used in the system is composed of OPA657, and the filter module that uses two low-pass Butterworth filter circuits is cascaded to form a fourth-order, low-pass Butterworth filter. After the detection signal is conditioned by the hardware, it can ensure that the analog voltage signal input to the acquisition chip has smooth and stable characteristics. The processed received signal is subjected to ADC sampling, and the detection data are collected and further sent to the PC.

To meet the needs of the practical application, the amplifying circuit needs to have a sufficiently high magnification and bandwidth. OPA657 has the characteristics of 1.6 GHz gain bandwidth product, low-voltage noise of $4.8\;\mathrm{nV}/\sqrt{\mathrm{Hz}}$, etc., [30] through the ratio of the reverse input resistance *R*_1_ and feedback resistance *R_f_* to control the gain change. There is a simplified linear relationship between the (*R*_1_ + *R_f_*)/*R*_1_ ratio and the magnification of the OPA657 operational amplifier.

Going through amplifier OPA657, the received signal contains high-frequency clutter and noise, so the design uses the LT1568 chip to cascade into a fourth-order active RC low-pass filter [31], and its cut-off frequency is 1 MHz. Whereas LT1568 is a low-noise, high-frequency active RC filter component, it can support a signal-to-noise ratio of more than 90 dB. It can also provide single-ended to differential signal conversion to achieve direct drive to the high-speed analog-to-digital converter.

The analog-to-digital converter is the core component of data acquisition, which affects the sampling accuracy, sampling rate, and data throughput of the entire system. The system uses the ADI analog-to-digital converter AD9226. This ADC has rich characteristics, e.g., single power supply, 12-bit precision, 65MSPS high-speed, 475 mW low power consumption, and 69 dB high signal-to-noise ratio [25].

### 2.4. System Workflow

**Figure 2:**
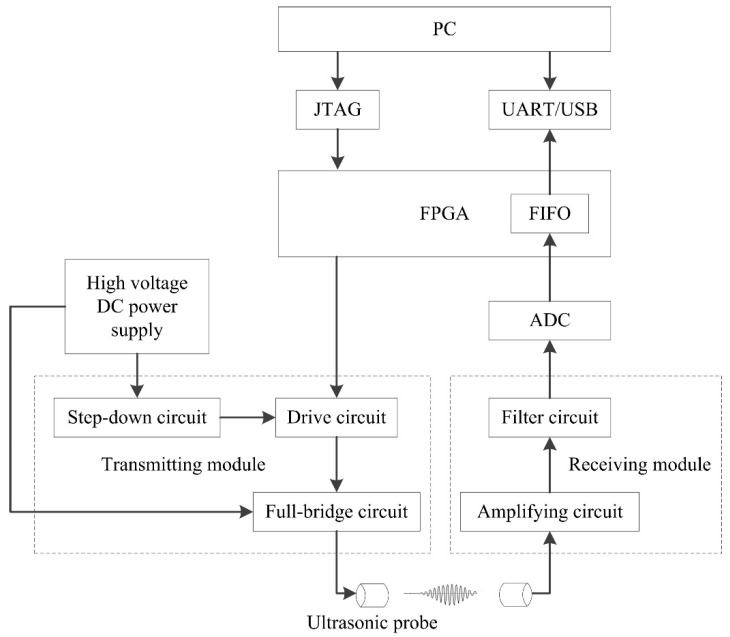
Block diagram of ultrasonic testing system.

The working process of the detection system is shown in Figure 2. After the system is powered on, it waits for the FPGA initialization to be completed and enters the waiting state for connection. When the relevant command parameters are inputted externally, the FPGA receives and parses the command, the FPGA controls the drive circuit to quickly open or close the MOS transistor of the full-bridge circuit according to the control program, and it generates square wave pulse with the set frequency to stimulate the probe to operate. The high-voltage DC power supply provides high voltage for the full-bridge circuit and, at the same time, provides the required operating voltage for the drive circuit through the step-down module. The receiving circuit amplifies and filters the transmitted ultrasonic and obtains the digital signal of the detection waveform through the ADC. The collected data are transmitted to the PC through the First Input First Output (FIFO) via the UART or USB interface for subsequent processing and analysis.

## 3. Performance Analysis of the System Submodule

In ultrasonic detection systems, the analysis of system performance has many technical indices such as the work stability, repeatability of output signals, consistency between output, and theoretical signals. The input–output characteristics of the ultrasonic detection system can reflect the working performance. Using these indices of the input–output signals, theory and actual output are the means to evaluate the performance of each submodule in the system.

### 3.1. Pulse Transmitting Module Performance Assessment

**Figure 3:**
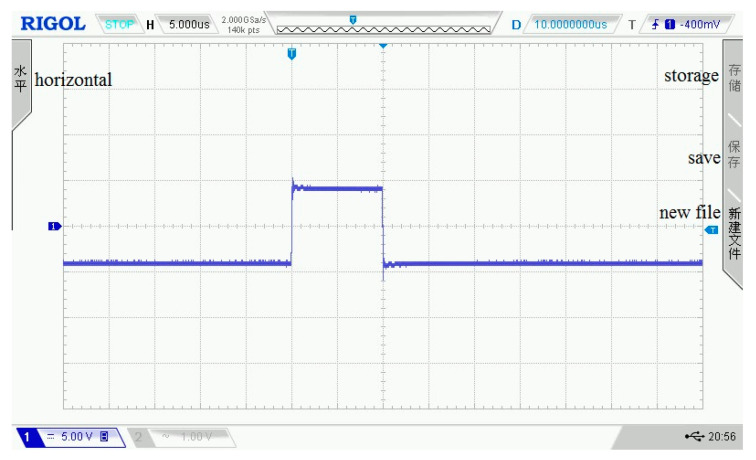
80 Vpp square wave pulse.

**Figure 4:**
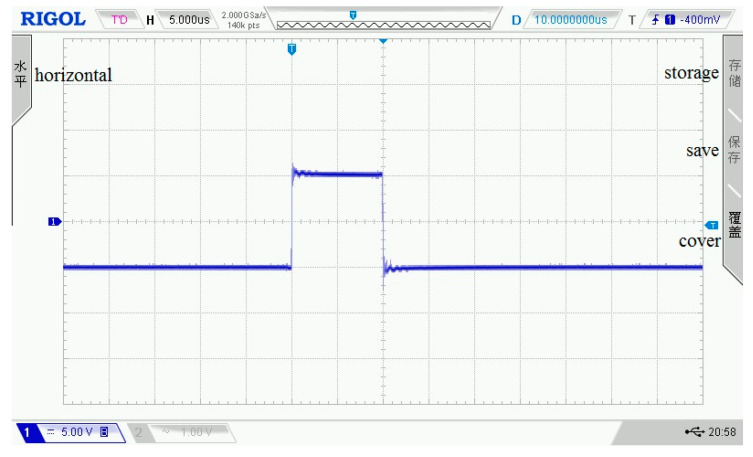
100 Vpp square wave pulse.

**Figure 5:**
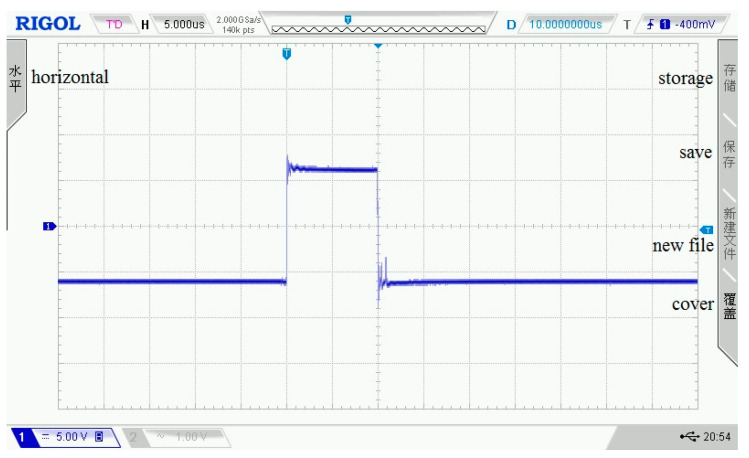
120 Vpp square wave pulse.

**Figure 6:**
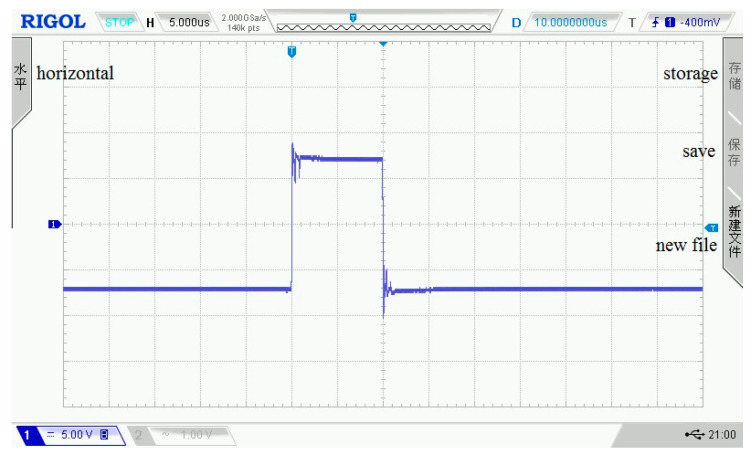
140 Vpp square wave pulse.

**Figure 7:**
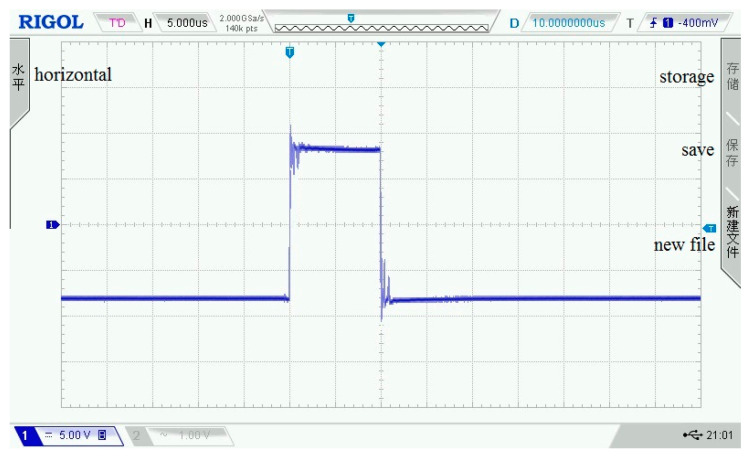
160 Vpp square wave pulse.

**Figure 8:**
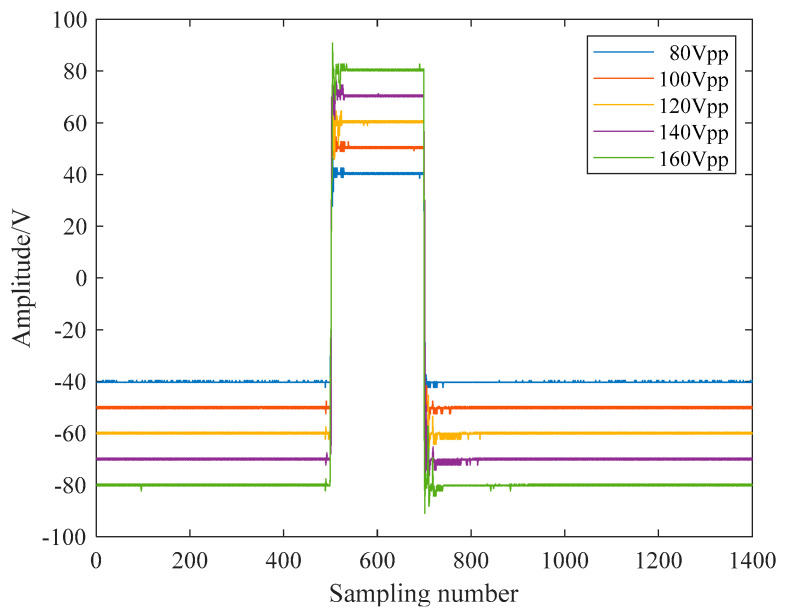
Square wave signals in the pulse emission circuit.

As shown in Figure 3, Figure 4, Figure 5, Figure 6 and Figure 7, 50 kHz square wave pulse signals with different amplitudes are generated by the transmitting circuit on the oscilloscope. In these figures, each grid of the abscissa is 5 μs, and each grid of the ordinate is 5 V. For displaying the output waveforms of the transmitting circuit completely on the oscilloscope, the actual signals are attenuated by 10 times. Their corresponding waveforms are plotted in Figure 8.

The amplitude and frequency of the theoretical square wave pulse signals are the preset values in the FPGA control program. The distortion of the output pulse signal is evaluated by comparing the square wave pulse waveform data and theoretical waveform data in actual work. Many assessment indices reflect the degree of similarity between the actual waveform and the theoretical waveform. Among them, the residual similarity and the Pearson correlation coefficient are employed relatively more frequently, and their calculations are simple [32,33].

The residual similarity calculation equation is given as follows, and its value range is [0, 1]. The closer to 1, the higher the similarity between the waveforms, and the smaller the difference.

(1) $$r=\frac{y_{r}-\left|y_{r}-y_{s}\right|}{y_{r}}$$

where *r* is the value of residual similarity, *y_s_* represents the actual output signal, and *y_r_* represents the theoretical output signal.

The Pearson correlation coefficient is used to calculate the correlation between two variables. The calculation expression is shown in Equation (2).

(2) $$p_{X,Y}=\frac{Cov\left(X,Y\right)}{\sigma_{X}\sigma_{Y}}$$

where *p* is the value of Pearson correlation coefficient, *Cov*(*X, Y*) is the covariance of the two variables *X* and *Y*, *σ_X_* is the standard deviation of *X*, and *σ_Y_* is the standard deviation of *Y*. The value range is between −1 and 1. The closer the correlation coefficient is to −1 or 1, the higher the correlation. The closer the correlation coefficient is to 0, the lower the correlation. The sign indicates the direction of the correlation. It is positive when one variable increases and the other variable also increases, and it is negative when one variable increases and the other variable decreases.

**Figure 9:**
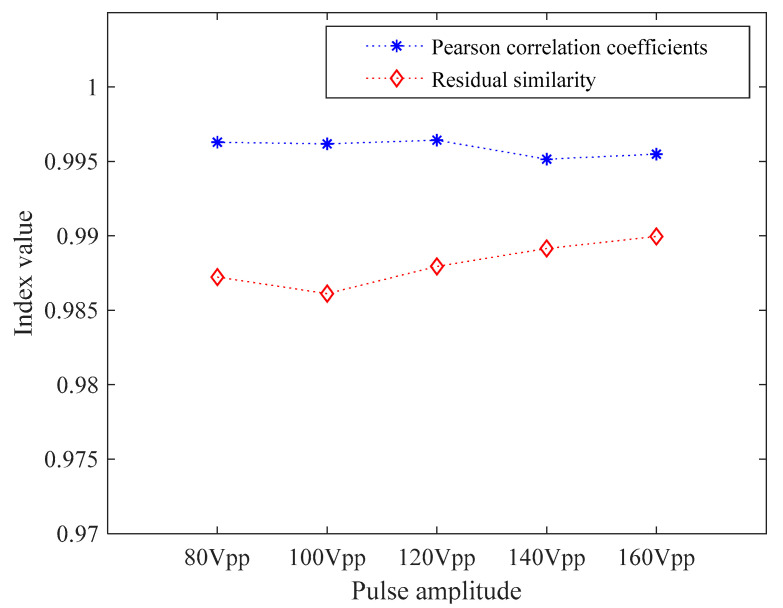
Residual similarity and Pearson correlation coefficients.

The residual similarity and Pearson correlation coefficient between theoretical and actual waveform data are calculated by Equations (1) and (2), and the calculation results are shown in the curve in Figure 9. It can be seen from Figure 9 that the residual similarity of the output square wave pulses under the five voltage conditions reached more than 0.98, and the correlation coefficients reached 0.99, indicating that the error between the actual output and the theoretical output of the transmitting circuit is small, which can meet the rated working conditions of the ultrasonic probe.

### 3.2. Receiving Module Performance Assessment

The signal receiving module mainly includes an amplifying circuit and a filter circuit. For evaluating the performance of the receiving module, the input of the circuit is the analog signal generated by the signal generator. By analyzing the magnification of amplified output signals and the filter bandwidth curve of filtered output signals, the performance of the module can be evaluated.

**Figure 10:**
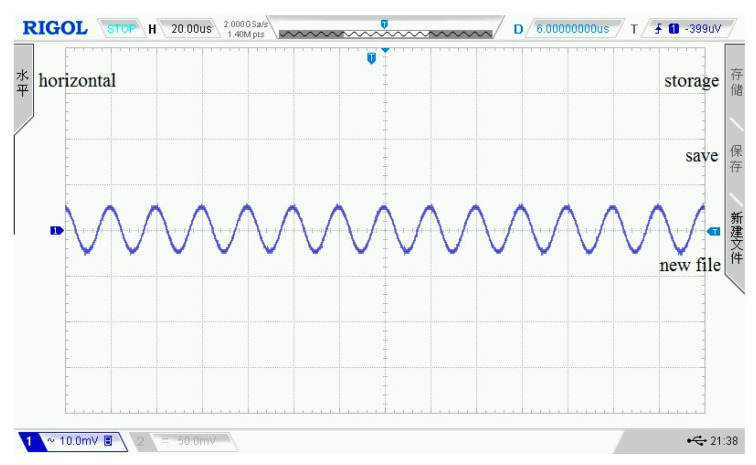
The input signal of the amplifier circuit.

**Figure 11:**
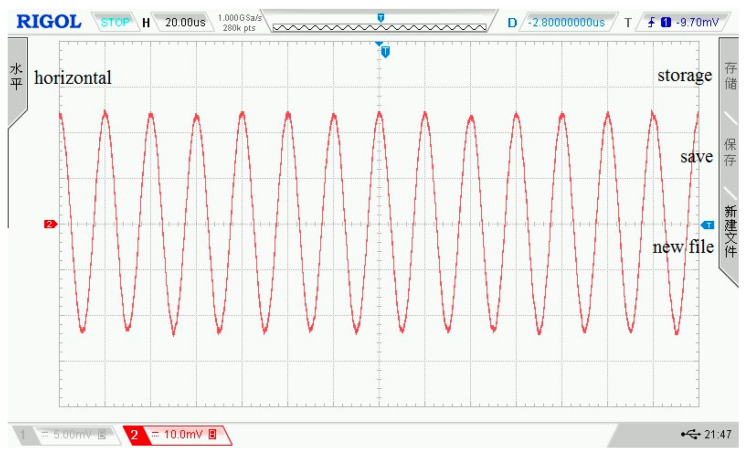
The output signal of the amplifier circuit.

In the assessment experiment of the amplifier circuit, the input signal amplitude is ±5 mV, the frequency is 50 kHz, and the output impedance is 50 Ω. Under the condition that the resistance ratio is 10, the input and output waveforms of the amplifier circuit on the oscilloscope are shown in Figure 10 and Figure 11. In the two figures, each grid of the abscissa is 20 μs, and each grid of the ordinate is 10 mV.

**Figure 12:**
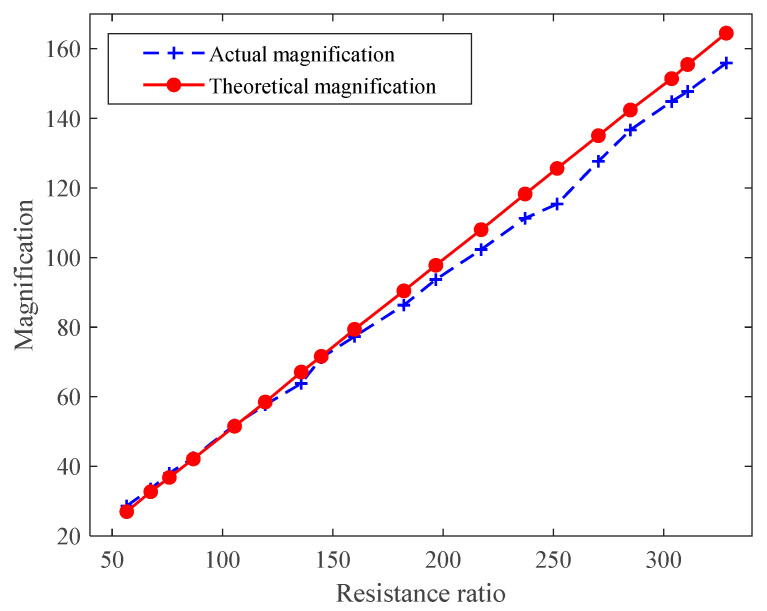
Test curve of OPA657 performance.

The magnifications measured at different ratios are shown in Figure 12. In Figure 12, there is a very small error between the actual measurement amplification curve and the theoretical amplification curve, which can provide sufficient gain for the detection signal.

**Figure 13:**
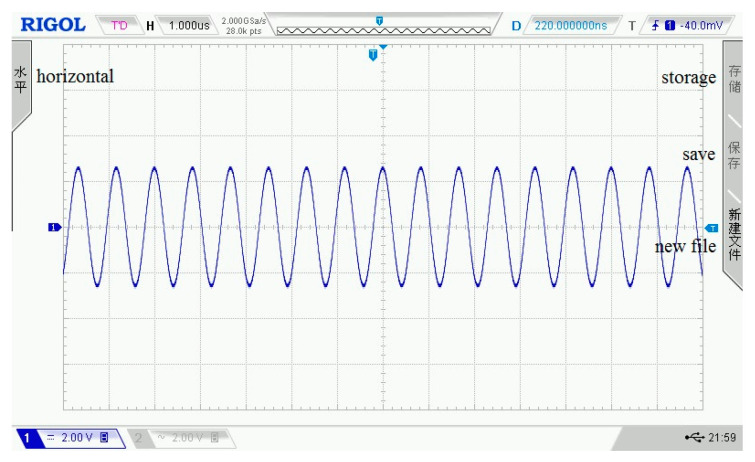
The input signal of the filter circuit.

**Figure 14:**
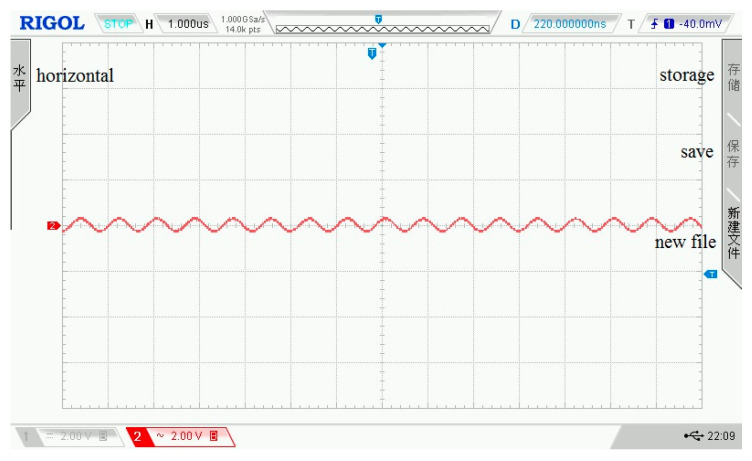
The output signal of the filter circuit.

**Figure 15:**
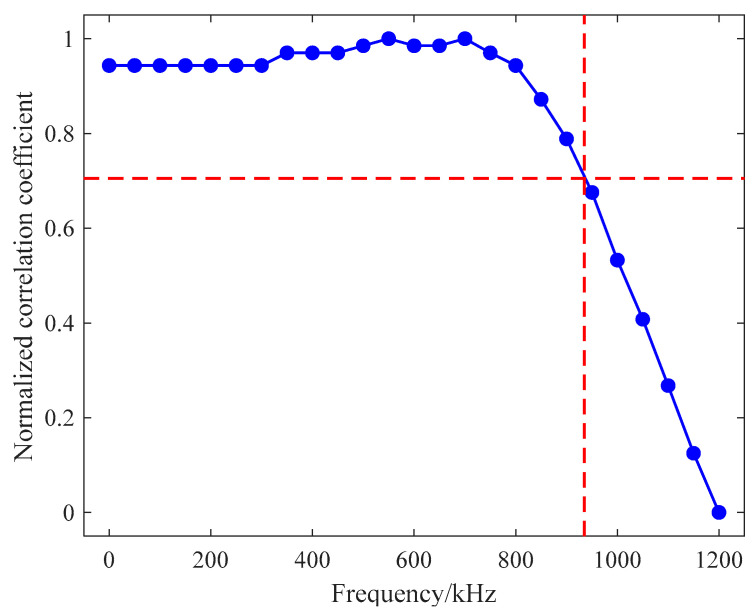
Test curve of filter bandwidth.

The sinusoidal signals with a frequency range of 0.1 kHz–1.2 MHz and amplitude of ±2.5 V serve as input to the filter circuit, and its output signals are collected. The input signal frequency of Figure 13 is 1.2 MHz, and the output waveform of the filter circuit is shown in Figure 14. In the two figures, each grid of the abscissa is 20 μs, and each grid of the ordinate is 10 mV. When the frequency of the input signal exceeds the cutoff frequency, its amplitude will be greatly attenuated, so as to achieve signal filtering. Figure 15 shows the amplitude of the normalized output signal. This curve shows that as the signal frequency increases, the signal amplitude decreases significantly when it approaches the cutoff frequency. At a frequency around 935 kHz, the amplitude of the output signal attenuates at 3 dB.

## 4. System Output Performance Analysis

**Figure 16:**
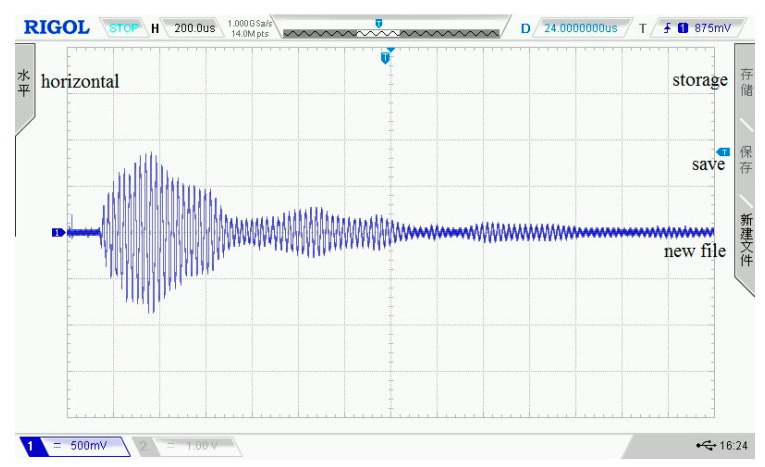
The single cycle ultrasonic signal.

**Figure 17:**
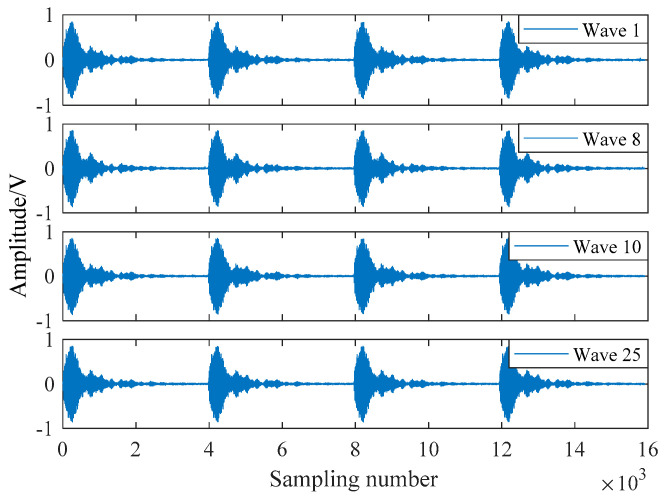
Four received waveforms.

In the working process of each submodule of the detection system described in this paper, it can be known from their assessment indices and test curves that each submodule can meet the design requirements and work stably. To analyze the overall output performance of the presented ultrasonic detection system, the system uses air as the propagation medium to carry out ultrasonic transmitting and receiving test experiments. The relevant parameter settings of the experimental environment are shown in Table 1. In the same experimental environment, each test obtains a received waveform data with four consecutive cycles, and a total of 40 waveform data are collected. The oscilloscope is used to display the waveform of ultrasonic transmitted in the air, where Figure 16 shows the waveform of one cycle. According to 40 received waveform data, the output performance assessment work is executed. In Figure 17, four waveforms are randomly chosen to display their data.

RMSE is used for the measure of differences between the variables. The higher value of RMSE implies the greater difference between the data set. In particular, the expression is shown in Equation (3).

(3) $$RMSE=\sqrt{\frac{1}{n}\overset{n}{\underset{i=1}{\sum }}{\left(X_{i}-Y_{i}\right)}^{2}}$$

where *X_i_* represents the value of the variable *X* at the *i*-th sampling point, *Y_i_* represents the value of the variable *Y* at the *i*-th sampling point, and *n* represents the number of sampling points.

The magnitude-squared coherence estimation is a frequency domain function, and its value is between 0 and 1, which represents the corresponding relationship at each frequency. Its expression is shown in Equation (4).

(4) $$C_{X,Y}=\frac{P_{XX}{\left(f\right)}^{2}}{P_{XX}\left(f\right)P_{YY}\left(f\right)}$$

where *C_X,Y_* is the magnitude-squared coherence, *P_XX_*(*f*) is the power spectral density function of *X*, *P_YY_*(*f*) is the power spectral density function of *Y*, and *P_XY_*(*f*) is the cross power spectral density function of *X* and *Y*.

**Figure 18:**
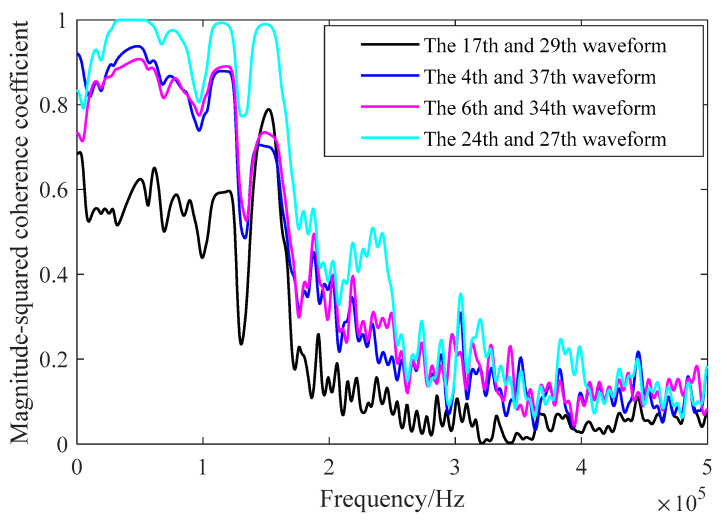
Analysis of waveform magnitude-squared coherence.

Table 2 lists four pairs of detection waveforms, which are the maximum, median, and minimum values of the Pearson correlation coefficient and RMSE. It can be seen from the two index values that there is a big difference between the 17th and 29th detection waveforms, while the 24th and 27th detection waveforms have the highest similarity. The magnitude-squared coherence is used as the index to analyze the waveforms in Table 2, and the results are shown in Figure 18. The Pearson correlation coefficient between the 24th and 27th waveforms is the largest and the RMSE is the smallest. The magnitude-squared coherence is also the largest, and the similarity between the waveforms is the highest. From the magnitude-squared coherence estimate waveform diagram, it can be seen that the magnitude-squared coherence values in the 0–160 kHz frequency band are larger. The noise and higher harmonics in the higher-frequency part of the waveform will increase, and magnitude-squared coherence will be reduced. The mean value of the magnitude-squared coherence of 40 test signal data is 0.8670.

**Figure 19:**
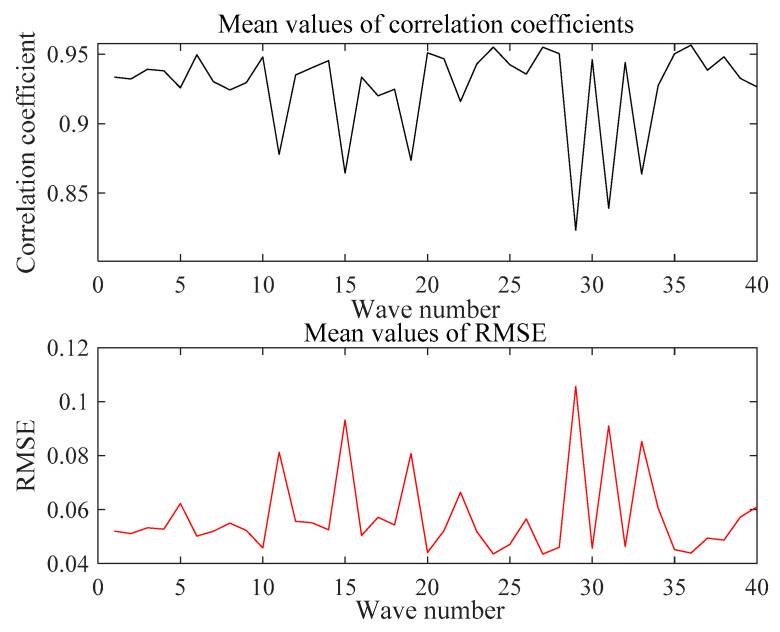
Average values of Pearson correlation coefficient and average values of RMSE.

In this paper, the Pearson correlation coefficient and RMSE between each test signal waveform are calculated in the experiment. In the received waveforms under the same test conditions, the maximum Pearson correlation coefficient is 0.9991, the minimum RMSE is 0.0069, the median Pearson correlation coefficient is 0.9459, and the median RMSE is 0.0564, but the minimum Pearson correlation coefficient is 0.7032 and the maximum RMSE is 0.1405. The average Pearson correlation coefficient and RMSE of 40 received waveforms are counted, and the values are 0.9265 and 0.0575, respectively. The average values of the Pearson correlation coefficient and RMSE are shown in Figure 19. Each waveform has a larger Pearson correlation coefficient and a smaller RMSE with other waveforms, which proves that the system is stable. Due to the deviation of the response time of the piezoelectric transducer during the test, the received waveforms have a certain phase difference, which makes the waveform Pearson correlation coefficient and RMSE values fluctuate.

## 5. Conclusions

Aiming at the demand for nondestructive testing of concrete structures, a high-performance, convenient, and low-cost ultrasonic testing system is proposed in this paper. This system uses FPGA as the control core and includes the pulse transmitter module, the signal conditioning, and the acquisition modules. The designed system can achieve adjustable output under certain performance indices requirements and results in outstanding characteristics, e.g., strong expansibility, high flexibility, simple and convenient structure, and stable operation. Under the working conditions set up in this paper, the performance of the system has been evaluated and analyzed. Based on the experimental data, Pearson correlation coefficient, residual similarity, root-mean-square error, and magnitude-squared coherence coefficient are selected as the assessment indices, and the working curves of the submodules are visualized to evaluate the performance of the system. The assessment result indicates that the system can work stably long-term at the rated frequency. In this paper, not only the designed and developed hardware part of the ultrasonic testing system, but also the evaluated performance of the system measured by experiment and assessment indices are exhibited, which provides a feasible scheme for the system performance assessment, especially for the experimental systems regarding concrete structure monitoring and detection.

It has been shown that multi-channel ultrasonic detection systems have been widely used in medical and urban monitoring fields [34,35]. It will be a valuable research topic to build a multi-channel ultrasonic detection system and carry out data fusion to achieve concrete internal detection images. In our future work, we will construct a theoretical model of the ultrasonic detection system. Then, we will be able to analyze and evaluate the performance of the presented ultrasonic detection system from the perspective of comparing the output of the theoretical model [36] with the output of the actual system.

## Figures and Tables

**Table 1:** System parameters setting in the experimental environment.

Experimental Parameters	Parameter Values
Pulse amplitude	±50 V
Pulse width	10 μs
Pulse interval	3.99 ms
Sampling frequency	1 MHz
Transducer center distance	45 mm
Magnification	46 dB

**Table 2:** Estimation results of the waveform analysis.

Wave Number	Wave Number	Pearson Correlation Coefficient	RMSE	Conclusion
17	29	0.7032	0.1405	The smallest Pearson correlation coefficient, the largest RMSE
4	37	0.9459	0.0532	Pearson correlation coefficient is the median
6	34	0.9462	0.0564	RMSE is the median
24	27	0.9991	0.0069	The largest Pearson correlation coefficient, the smallest RMSE

## Data Availability

Not applicable.
